# Glutamate concentration of medial prefrontal cortex is inversely associated with addictive behaviors: a translational study

**DOI:** 10.1038/s41398-024-03145-x

**Published:** 2024-10-12

**Authors:** Hui Zhou, Tiantian Hong, Xi Chen, Conghui Su, Binyu Teng, Wan Xi, Jean Lud Cadet, Yihong Yang, Fengji Geng, Yuzheng Hu

**Affiliations:** 1 Department of Psychology and Behavioral Sciences, Zhejiang University, Hangzhou, 311100 China; 2 The State Key Lab of Brain-Machine Intelligence, Zhejiang University, Hangzhou, 311100 China; 3 Department of Psychiatry, Harvard Medical School, Boston, MA 02115 USA; 4 Molecular Neuropsychiatry Research Branch, National Institute on Drug Abuse, Intramural Research Programs, National Institutes of Health, Baltimore, MD 21224 USA; 5 Neuroimaging Research Branch, National Institute on Drug Abuse, Intramural Research Programs, National Institutes of Health, Baltimore, MD 21224 USA; 6 Department of Curriculum and Learning Sciences, Zhejiang University, Hangzhou, 311100 China; 7 MOE Frontiers Science Center for Brain Science & Brain-Machine Integration, Zhejiang University, Hangzhou, 311100 China; 8 Key Laboratory of Novel Targets and Drug Study for Neural Repair of Zhejiang Province, School of Medicine, Hangzhou City University, Hangzhou, 310015 China

**Keywords:** Addiction, Human behaviour

## Abstract

In both preclinical and clinical settings, dysregulated frontostriatal circuits have been identified as the underlying neural substrates of compulsive seeking/taking behaviors manifested in substance use disorders and behavioral addictions including internet gaming disorder (IGD). However, the neurochemical substrates for these disorders remain elusive. The lack of comprehensive cognitive assessments in animal models has hampered our understanding of neural plasticity in addiction from these models. In this study, combining data from a rat model of compulsive taking/seeking and human participants with various levels of IGD severity, we investigated the relationship between regional glutamate (Glu) concentration and addictive behaviors. We found that Glu levels were significantly lower in the prelimbic cortex (PrL) of rats after 20-days of methamphetamine self-administration (SA), compared to controls. Glu concentration after a punishment phase negatively correlated with acute drug-seeking behavior. In addition, changes in Glu levels from a drug naïve state to compulsive drug taking patterns negatively correlated with drug-seeking during both acute and prolonged abstinence. The human data revealed a significant negative correlation between Glu concentration in the dorsal anterior cingulate cortex (dACC), the human PrL counterpart, and symptoms of IGD. Interestingly, there was a positive correlation between Glu levels in the dACC and self-control, as well as mindful awareness. Further analysis revealed that the dACC Glu concentration mediated the relationship between self-control/mindful awareness and IGD symptoms. These results provide convergent evidence for a protective role of dACC/PrL in addiction, suggesting interventions to enhance dACC glutamatergic functions as a potential strategy for addiction prevention and treatment.

## Introduction

Although the initial reinforcement effects are different, both substance use disorders (SUDs) and internet gaming disorder (IGD) are characterized by recurrences of compulsive behaviors that sustain individuals’ use of various substances or playing games despite harmful consequences [1, 2]. Previous studies have indicated that dysregulated frontostriatal circuits contribute to the development of addictive behaviors, both in preclinical models [3–5] and clinical investigations [6–8]. Specifically, using resting-state functional connectivity (rsFC), we previously demonstrated that the rsFC strength between dorsal anterior cingulate cortex (dACC) and ventral striatum was inversely associated with DSM-IV diagnostic symptoms concerning behaviors relevant to loss-of-control over drug use [7]. This relationship was further confirmed by using an animal model, where a negative correlation was observed between compulsive drug taking behavior and rsFC between prelimbic cortex (PrL; the rodent homolog of human dACC [9]) and ventral striatum [10]. Relatedly, lower PrL excitability at the cellular level in a rodent addiction model has been documented [3]. Previous human studies also found that error-related dACC *hypoactivity* predicts cocaine relapse [11], while error-related dACC *hyperactivity* is observed in individuals who have successfully maintained long-term cocaine abstinence [12].

Considering that the ventral striatum receives glutamatergic inputs from prefrontal cortex and that glutamate is the major excitatory neurotransmitter modulating task activation [13, 14], it is reasonable to conjecture that the regional activation of PrL/dACC influenced by glutamate concentration may also regulate addictive behaviors. The above findings suggest that the glutamate concentration of dACC/PrL may change across different stages of addiction. However, the specific alterations in glutamate concentration within the PrL/dACC at a systems level and their relationships with addiction have yet to be fully delineated.

To gain deeper insights, a translational approach that combines data from humans and animal models with complementary assessments would yield a more complete understanding of the impact of regional glutamate concentration on addictive behaviors. This is because that preclinical studies can be well controlled for many potential confounding factors, such as comorbidity that are often seen in human participants with SUD, but assessing high-order cognitive functions in animals poses significant challenges. In contrast, human studies can link findings from preclinical models, such as associations between neural plasticity and relevant behaviors, as well as extensive psychologic assessments.

For the psychologic mechanism of addiction, previous studies have highlighted self-control and mindful awareness as two factors that potentially modulate addictive behaviors [15–17]. Self-control refers to the ability to regulate one’s thoughts, emotions, and behaviors in the pursuit of long-term goals. Previous human research has identified that adolescents with Internet addiction displayed lower level of self-control compared to those without Internet addiction [18]. Additionally, self-control has been found to be negatively correlated with online game addiction [16]. In a previous study, we also found a positive correlation between self-control and the rsFC of the dACC - ventral striatum pathway [17]. Mindful awareness, on the other hand, is the ability to attend to and be fully present in the current moment with an open and nonjudgmental attitude [19]. By training participants to increase awareness of automaticity and craving, mindfulness intervention was considered as a behavioral strategy to de-automatizes addictive behavior, possibly by increasing connectivity between top-down and bottom-up brain networks implicated in addiction (e.g., frontostriatal circuitry) [15]. Indeed, previous meta analyses indicated that different types of mindfulness meditation could increase the activation of dACC region [20].

**Fig. 1:**
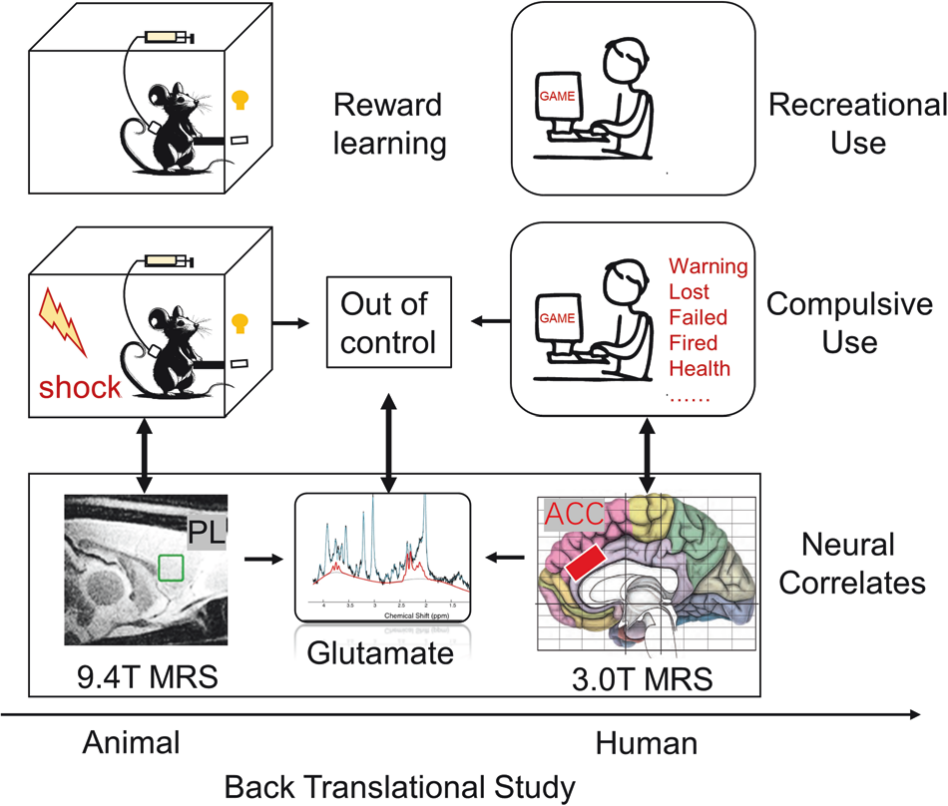
A translational approach that combines human and animal models with complementary assessments to yield a more complete understanding of the role of regional excitability in addiction. Rats were trained to self-administer substance, followed by a phase of foot shock punishment. Persistent lever pressing for drug under punishment is regarded as compulsive use with similar psychological and neural processes underlying human addictive behaviors such as Internet gaming disorder. With a magnetic resonance spectroscopy technique, the present study examined the prefrontal glutamate changes with a longitudinal design and investigated the translational implications with high-order psychological assessments in human subjects.

Although the dACC activation and functional connectivity were closely associated with both self-control and mindfulness awareness, the role of glutamate concentration of dACC region in the relationship between these cognitive functions and addiction has not been fully explored. To this end, we combined a preclinical model and human subjects to investigate the role of dACC/PrL glutamate concentration in addiction, using a magnetic resonance spectroscopy (MRS) technique (Fig. 1). A longitudinal rodent study with a model of stimulant addiction [21, 22] was conducted to assess the alterations of PrL glutamate concentration across different stages along the development into drug addiction, and then the relationships between PrL glutamate and compulsive drug seeking and taking behaviors were examined. Next, to translate our findings from the animal model to humans, we conducted a cross-sectional human study with multiple cognitive assessments to better understand the functional implications of the PrL/dACC glutamate concentration in regulating addictive behaviors.

Based on findings in previous human and preclinical studies [3–5, 7, 23], we hypothesized that 1) the glutamate concentration in rodent PrL would be decreased after substance use, and would negatively correlate with drug seeking and compulsive drug taking behaviors, 2) the human dACC glutamate concentration would negatively correlate with the severity of IGD, but positively correlate with mindful awareness and self-control, and 3) the dACC glutamate concentration would mediate the relationship between the severity of IGD and self-control, as well as mindful awareness.

## Methods and materials

### Self-administration rat experiment

Animal procedures were approved by the Animal Care and Use Committee of the National Institute of Drug Abuse Intramural Research Program and followed the Guide for the Care and Use of Laboratory Animals (ISBN 0-309-05377-3). Forty-one male Sprague-Dawley rats (Charles River Labs, Raleigh, NC, USA) were used in the animal experiment, and all rats received surgery at 14–16 weeks old and weighted 350–400 g before surgery. The rats were randomly assigned to either a METH group (*n* = 26) or a saline control group (SAL, *n* = 15). The sample size was consistent with that of a previous study, which achieved significant results [10]. Based on this, we anticipate that the sample size for the current study will be sufficient. During the experiment, each rat underwent three phases, including a 20-day self-administration (SA) training phase, a 5-day foot shock (FS) punishment phase, and a 30-day abstinence phase in which two cue-reactivity tests were conducted (please refer to Supplementary methods for details). Glutamate concentration was measured at three time points, before SA training (S1, baseline), after SA training (S2), and after a 5-day FS punishment (S3).

During the SA phase, rats stayed in a chamber equipped with two levers: an active lever and an inactive lever. Pressing the retractable active lever triggered an infusion pump, delivering either METH or saline. Pressing the inactive lever had no reinforced consequences. A 5-s compound tone-light cue was paired with each infusion.

During the punishment phase, pseudorandom FS lasting 0.5 s were introduced alongside METH SA, occurring with half of the reinforced lever-presses. The intensity of the foot shocks varied over the 5 days in a predetermined fixed order (0.18, 0.24, 0.3, 0.3, and 0.3 mA) [21, 22]. It is important to note that a subset of eight METH rats and four SAL rats underwent a longer FS period than the standard 5 days. These rats were simply part of the same batch in which all rats underwent a total of 11 days of FS. Notably, the METH rats in this group were not necessarily the resistant ones. The Glutamate level of rats that later had longer FS period did not significantly differ from those had standard 5-day FS period, neither before SA training nor after SA training (METH: before SA: *t* (24) = 0.813, *p* = 0.424; after SA: *t* (24) = 1.196, *p* = 0.243, please see Supplementary Fig. 1 for infusion performance of these rats that underwent a longer FS period). Consequently, the data collected from these rats were not included in any analyses pertaining to the data collected after the FS period, resulting 18 METH rats and 11 SAL rats in these analyses.

**Fig. 2:**
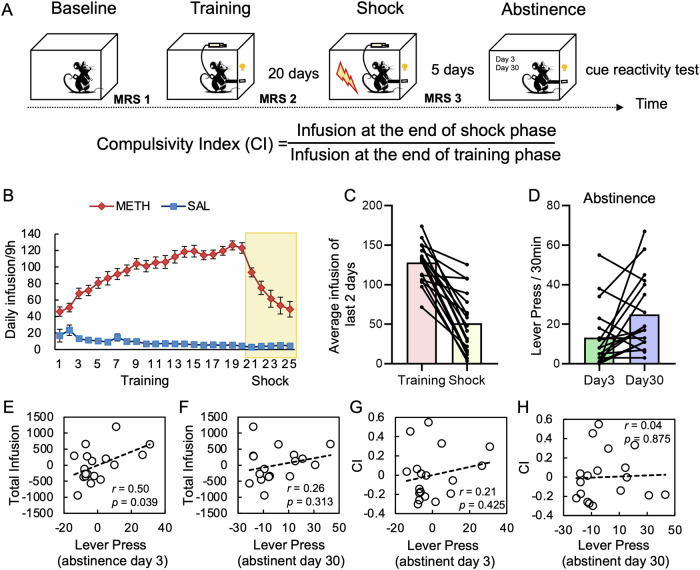
Animal experiment design and behavioral results. **A** In the longitudinal design, two groups of rats were trained to self-administer saline (SAL) or methamphetamine (METH), followed by a shock punishment phase and two cue-reactivity sessions. **B** The METH group significantly increased their drug intake during the training phase and decreased when foot shock was introduced, whereas the SAL group remained at a low level of saline infusion. **C** Infusion amount at the ends of the training and shock phases (average infusions of last two training days and last two foot-shock days, respectively). **D** Active lever press of the cue-reactivity tests at abstinent day 3 and day 30. **E**, **F** Partial correlations between total infusion of METH (summing up daily infusions during training and foot shock sessions) and active lever press of the cue-reactivity tests at day 3 and day 30. Each data point indicates a rat from the METH group. **G**, **H** Partial correlations between CI (compulsivity index) and active lever press of the cue-reactivity tests at day 3 and day 30. Each data point indicates a rat from the METH group. The number of inactive lever press at the cue-reactivity session at abstinent day 3 was included as a covariate in the partial correlational analyses. The error bars represent the standard error of the mean. SAL saline, METH methylamphetamine.

During the abstinence phase, cue-presentation (extinction) tests were conducted on day 3 and day 30 of the abstinence period, each lasting for 30 min (Fig. 2A). During these sessions, the rats were placed back into the SA chambers, where pressing the previously active lever no longer resulted in the delivery of METH or saline. Instead, pressing the lever solely triggered the presentation of the tone-light cue that was previously associated with METH/saline infusions.

### Human IGD experiment

#### Subjects

In the human MRS experiment, sixty-nine participants were recruited by online advertisement from Zhejiang University. This experiment was approved by the Ethic Committee of Zhejiang University. Informed consent was obtained from each participant before the study, and all participants were compensated for their time dedication. The severity of IGD was assessed by Internet Addiction Test (IAT) [24, 25], and DSM-5 based IGD scale (abbreviated as DSM) [2, 26]. The trait mindfulness of participants were measured by the Chinese version of five-facet mindful questionnaire [19, 27], which consists of five subscales: 1) *Observing*. 2) *Describing*. 3) *Acting with awareness*. 4) *Non-judging of inner experience*. 5) *Non-reactivity to inner experience*. *Acting with awareness* was used as a measure of mindful awareness in the present study. The level of self-control, problematic usage of social media, and the anxiety and depression levels of participants were assessed with Self-control scale [28, 29], Social media disorder Scale (SMD) [2, 30], and Brief Symptom Inventory (BSI) questionnaire [31], respectively (please refer to Supplementary methods for questionnaire details). No subject reported clinically diagnosed depression or anxiety disorder. Regarding illegal drugs, as they are strictly prohibited in China and the likelihood to use them among college students is extremely low, and nobody would claim the use of illegal drugs due to legal concerns. Therefore, we did not survey their use.

#### Data quality control

Five participants were excluded due to the absence of MRS data. To ensure the inclusion of high-quality data, we applied the following exclusion criteria: Cramer-Rao lower bounds (CRLB) of dACC greater than 0.07 (*n* = 8), dACC gray matter proportion, white matter portion or cerebrospinal fluid exceeds three standard deviations above or below the mean (*n* = 2). These criteria were used to ensure data quality and minimize confounding factors. The final human sample after data quality control consisted of 54 participants (age = 22.33 ± 2.42, range 18–28, 35 males) (please refer to Supplementary Table 1 for detailed demographic information).

### MRI experiments

#### Image acquisition and preprocessing

Animal MRI data were acquired on a Bruker BioSpin 9.4T scanner (Bruker Medizintechnik, Karlsruhe, Germany). MRS study was performed on the prelimbic cortex (PrL, voxel size 2.5 × 2 × 2 mm^3^) using the home-optimized short TE PRESS sequence (TE/TR = 10/2000 ms). The voxel sizes and locations were referred to a previous study [32] with minimal inclusion of CSF and white matter. An interactive 3D shimming was performed for each targeted region before MRS scans with a criterion of water linewidth <14 Hz. The scans were acquired with 480 averages, totaling 16 min. In order to minimize the voxel shifts caused by chemical shift displacement, the center frequencies of the location modules were set to 2.3 ppm, aligning with the interested neurotransmitters. The water suppression was achieved using a variable pulse power and optimized relaxation delays water suppression (VAPOR) scheme [33] using Gauss shape pulse with a suppression bandwidth of 200 Hz. The unsuppressed water signal was also acquired with the same sequence for each region. RF pulses were turned off in the VAPOR module to preserve the same gradient effect as water-suppressed acquisition. The center frequencies of the location modules were set to water frequency (4.7 ppm). The water suppressed scans were saved with every 8 averages for phase and frequency corrections.

Human MRI data were collected using a 3T Siemens Prisma scanner equipped with a 20-channel coil. To measure the glutamate concentration, we acquired single-voxel proton MRS data from bilateral dACC (15 × 21 × 26 mm^3^), and a control region was placed at the primary visual cortex (V1, 15 × 21 × 26 mm^3^, Supplementary Fig. 2). The dACC voxel was aligned with the inferior face of the anterior corpus callosum, with an angle tangential to the corpus callosum, and placed in the center of two hemispheres. The V1 voxel was placed vertically in the center of the occipital lobe, ensuring that the entire voxel remains within the occipital lobe region. We used a MEGA-PRESS sequence (TE/TR = 68/2000 ms) with 72 averages to measure the glutamate concentration in both regions. The auto-shimming method was performed prior to the MRS scans. Additionally, we employed a VAPOR module [33] to achieve water suppression. Spectra of water were also acquired from the same VOI using the same MEGA-PRESS sequence, with a single average of water spectra.

#### MRS data preprocessing

The preprocessing of MRS data of animal was performed using MRS toolkit FID-A [34]. Phase and frequency corrections were applied on metabolite spectra with 8 averages per block before summed together. The original combined metabolite spectra were processed and quantified in LCModel [35] version 3.6-1H. The basis spectra of metabolites were simulated with the same experimental parameters in VeSPA [36] and an experiment acquired macromolecule spectrum from a whole brain voxel was included in the basis sets to account for the baseline in the short TE condition. The combined water spectra were used for eddy current corrections and concentration references.

MRS data from human participants were preprocessed using the MRS toolkit FID-A [34] and quantified using LCModel [35] version 3.6-1H. LCModel fits the experimental spectrum in the frequency domain by combining a group of basis sets, each representing the frequency spectrum of a specific metabolite/molecule, with a baseline to compensate for any signals that are not accounted for by the basis sets. The reliability of the measurement was assessed using the Cramer-Rao lower bounds (CRLB), with a criterion of 7% used in this study to reject low-quality spectra. The MRS signal is affected by differences in proton densities, T1 and T2 relaxation times in gray matter (GM), white matter (WM), and cerebrospinal fluid (CSF). Therefore, the volume fractions of different tissue types were calculated by segmenting T1 images using Gannet [37]. The water signal was then corrected by tissue fractions with different proton density according to a previous study [38]. T1 and T2 values of Glutamate in WM (T1 = 1170 ms, T2 = 124 ms) and GM (T1 = 1270 ms, T2 = 135 ms) [39, 40] were used for a global relaxation correction to obtain absolute quantification in millimolar (mM), assuming no contribution to Glu MR signal from CSF. The proton density ratio of WM to GM of Glu (0.5) [41–43] was used to calculate the Glu concentrations in GM. The equation [14] for correction calculations is outlined as follows:

$${({S}_{{met}}/{S}_{{wat}})}_{{corr}}={({S}_{{met}}/{S}_{{wat}})}_{{uncorr}}\cdot \frac{({WM} \% \cdot {f}_{{wat}{\rm{\_}}{WM}}+{GM} \% \cdot {f}_{{wat}{\rm{\_}}{GM}}+{CSF} \% \cdot {f}_{{wat}{\rm{\_}}{csf}})}{({WM} \% \cdot {f}_{{met}{\rm{\_}}{WM}}+{GM} \% \cdot {f}_{{met}{\rm{\_}}{GM}})}$$

Where *S* is the metabolite/water signal; WM%, GM% and CSF% are the tissue percentages; f is the sensitivity factor of metabolite/water in a certain tissue type.

### Statistical analyses

#### The animal experiment

For animals, the escalation of drug intake during the SA phase and the reduction of drug intake during the punishment phase were analyzed. One-way repeated analyses of variance (ANOVA) were conducted to test whether the average infusion of METH or SAL was increased during the SA training phase, and decreased after the FS was introduced (from the last day of SA training to the last day of FS). The number of active lever press during cue-reactivity tests were used as measurements to characterize the craving-induced drug seeking. In addition, infusions of METH on the last two FS days were averaged and then divided by the average of infusions on the last two training days to create a “compulsivity index (CI)” to assess compulsive drug taking behavior. Drug intake dosage was calculated by summing up daily infusion dosage during the whole SA training and FS period, serving as an index for total infusion. Partial correlations were performed to investigate the relationships between drug seeking and drug intake dosage, as well as the compulsive drug taking behavior, with responding on inactive lever-press on day 3 of the abstinence phase included as a covariate to control for individual differences in lever press tendency.

Mixed ANOVAs, with Group (METH vs. SAL) as the between subject factor and Session (S1, 2, and 3) as the within subject factor was performed to test the group difference of glutamate concentration of PrL across different stages of the addiction trajectory. Independent two-sample *T*-tests, or paired *T*-tests were employed to examine the glutamate concentration difference between groups and within groups. The Bonferroni correction was used to adjust for multiple comparisons; specifically, an uncorrected *p* < 0.01 was considered as significant when assessing glutamate concentration changes (5 comparisons, corrected *p* < 0.05, see below). To examine the relationship between glutamate concentration of PrL and drug taking, as well as drug seeking behaviors, partial correlation analyses were conducted, with inactive lever pressing on day 3 of the abstinence phase as a covariable.

#### The human experiment

In contrast to rats, human participants are more heterogeneous and more covariables were included. Partial correlation analyses were conducted to measure the relationship between the corrected glutamate concentration of dACC and addictive symptoms, as well as mindful awareness and self-control scores. In the current study, we treated DSM scores as a continuous variable to establish brain-behavioral relationship using individual difference. This approach aligns with the DSM-5’s recommendation to characterize IGD with multiple severity levels [2]. As social media overuse may confound with IGD, the SMD scale was measured and was included as a covariate in the partial correlation analyses, together with demographic information including age, gender, level of education. CRLB was also included as a covariate in the model to account for variations related with signal noise ratio, and tissue component of CSF of dACC was included as a covariate to further control for the tissue composition variations in human subjects. As for the analysis of the control region, 28 participants meeting the data quality criteria were included (age = 22.68 ± 2.68, 13 males).

The predicted mediation model was tested with Andrew F. Hayes’ SPSS macro PROCESS [44] by evaluating the three regression models, Y = cX + e_1_, M = aX + e_2_, Y = c’X + bM + e_3_. The significance of indirect effect was bootstrapped using 5000 bootstrap samples with replacement and a 95 percent bias-corrected CI that does not include zero was considered as significant.

## Results

### Behavior results of the animal model

Forty-one rats, randomly assigned to two groups, were trained to press a lever to obtain saline (SAL, n = 15) or methylamphetamine (METH, n = 26; 0.1 mg/kg/infusion) during a 20-day self-administration (SA) training phase (Fig. 2A). A 5-s tone-light compound cue was paired with the active (METH or SAL) lever but not with an inactive (control) lever. A subgroup of rats in the METH group (n = 18) went through a 5-day foot shock (FS) punishment phase. After the FS phase, there were two sessions of cue reactivity test (METH group, n = 18) at day 3 and 30 during which animals could press the previously drug-paired lever but no drug was delivered. As shown in Fig. 2B, rats in the METH group increased drug intake during the SA training phase (one-way ANOVA on daily infusion from day 1 to day 20, *F*_(5.48, 115.05)_ = 33.78, *p* < 0.001). When half trials of the drug SA were paired with FS during the subsequent 5-day punishment phase (Fig. 2B, day 21 to 25 with electric shock intensities of 0.18, 0.24, 0.3, 0.3, and 0.3 mA, respectively), rats in the METH group reduced their SA behavior and drug intake (repeated measure one-way ANOVA of infusions from the last SA day to the last FS day, *F*_(2.63, 44.64)_ = 22.81, *p* < 0.001). While every rat reduced drug taking when FS was introduced (paired T-test on the difference between average infusion of the last two SA training days and that of the last two FS punishment days, *t*_(17)_ = 8.02, *p* < 0.001), a large individual difference was seen (Fig. 2C). In the abstinence phase, the putative craving-induced drug seeking behavior was characterized by the number of active lever-presses in the two cue-reactivity tests (at day 3 and day 30), and a paired T-test revealed that rats in the METH group significantly increased their active lever pressing after prolonged abstinence (lever press on day 30 versus day 3, *t*_(17)_ = 2.37, *p* = 0.030, Fig. 2D). As some animals may press the active lever more frequently due to a general tendency to press any lever, responding on inactive lever-press at day 3 during the abstinence phase was used as a covariate when examining relationships between total infusion and drug seeking behavior. The number of active lever-press at day 3 was positively correlated with the amount of total infusion of METH during the training and shock phases (*r* = 0.50, *p* = 0.039, Fig. 2E), but such relationship was no longer significant after prolonged abstinence of 30 days (Fig. 2F). Additionally, neither active lever press at day 3 nor day 30 was correlated with CI (Fig. 2G, H), suggesting that these cue-reactivity tests capture a different aspect of addiction phenotype that is not manifested by compulsive drug taking under punishment.

### MRS results of the animal model

**Fig. 3:**
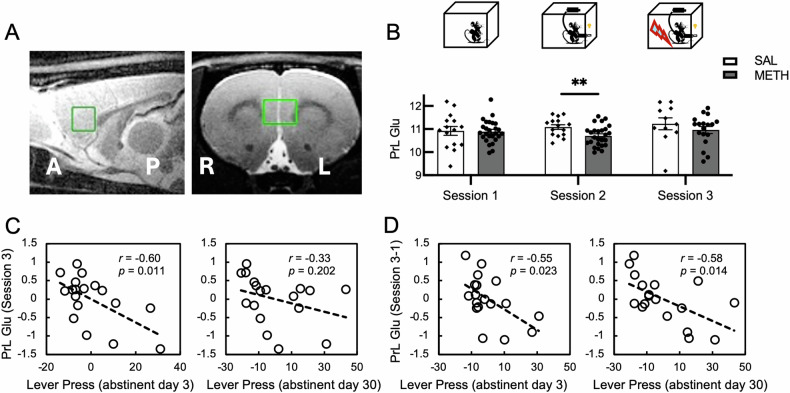
Glutamate concentration in PrL at different stages of the addiction model and changes of the glutamate concentration across different stages, as well as their relationship with drug seeking behavior. **A** The anatomic location of PrL where the MRS data were collected from. **B** Bar plots of glutamate concentration. The METH group exhibit significant lower level of concentration than the SAL group after the training periods. The error bar represents the standard error of mean. **C** Partial correlation between glutamate concentration (measured after the shock phase) and number of active lever pressing during the cue-reactivity sessions at abstinent day 3 or day 30. Each data point indicates a rat from the METH group. **D** Partial correlation between glutamate concentration change (concentration measured after the shock phase relative to that measured at baseline) and number of active lever pressing at abstinent day 3 or day 30. Each data point indicates a rat from the METH group. The pressing of inactive lever at the cue-reactivity session at abstinent day 3 was included as a covariate of confound in all partial correlations. **p < 0.01.

Next, we sought to examine the changes in glutamate concentration of PrL and how it was related to drug seeking behavior. MRS data were collected from a 2.5 × 2 × 2 mm^3^ volume of interest (VOI) in the PrL region (Fig. 3A). We first examined whether the glutamate concentration of PrL would display different trajectories of change between the two groups during the whole experiment. The glutamate concentration of each group essentially conforms to a normal distribution (Supplementary Table 2). A 2 (Group: METH vs SAL)-by-3 (MRS session 1, 2, and 3) ANOVA did not show any main effect of Group (*F*_(1, 27)_ = 1.28, *p* = 0.268) or Group-by-Session interaction (*F*_(2, 54)_ = 0.66, *p* = 0.522). Considering that the glutamate concentration may recover under the punishment phase, we further conducted a 2 (Group: METH vs SAL)-by-2 (MRS session 1, 2) ANOVA, and the results showed a marginally significant Group-by-Session interaction (*F*_(1, 39)_ = 3.57, *p* = 0.066). Nevertheless, our previous studies have identified that the glutamate of ACC in chronic cocaine users was significantly lower than healthy controls [45], and after an acute abstinence period, the Glx in the caudate-putamen was significantly lower in the monkeys of METH group than the control group [46]. Therefore, a difference in glutamate concentration in PrL between the two groups after training but not baseline was expected. To this end, independent two sample T-tests were conducted and rats in the METH group showed a lower level of glutamate concentration after training (session 2: METH - SAL, *t*_(39)_ = −2.74, *p* = 0.009, Fig. 3B). Further, paired T-tests also showed a lower level of glutamate after 20 days’ SA in the METH group, but this result did not pass the multiple comparison correction (paired t-test, SA - baseline, *t*_(25)_ = −1.79, one tailed *p* = 0.043, a *p*-value < 0.01 was considered to pass the correction for multiple comparisons across 5 t-tests). The glutamate concentration in the control group did not change after SA training, relative to that measured before training (paired t-test, SA - baseline, *t*_(14)_ = 0.99, one tailed *p* = 0.831). In addition, there is no difference in the level of glutamate between the two groups after FS (session 3: *t*_(27)_ = 0.95, *p* = 0.349). The two groups were not different at baseline measures (session 1: *t*_(39)_ = 0.15, *p* = 0.884), suggesting intake of METH is sufficient to decrease PrL glutamate and punishment may introduce a recovery. Next, we performed partial correlation analyses to examine the relationship between glutamate concentration and drug seeking behavior. As indicated above, the responding on the inactive lever at abstinence day 3 during the abstinence phase was used as a covariate. The PrL glutamate after FS (session 3) was found to correlate with drug seeking behavior at abstinent day 3 but not day 30 (Fig. 3C). In contrast, the changes of PrL glutamate concentration at session 3 relative to baseline (session 1), which may account for the cumulative effects of drug reward and shock punishment, correlated with drug seeking behavior at both abstinent day 3 and day 30 (Fig. 3D). In contrast, the correlation between glutamate concentration and CI was not significant (minimum *p* = 0.169).

### MRS results of human participants

**Fig. 4:**
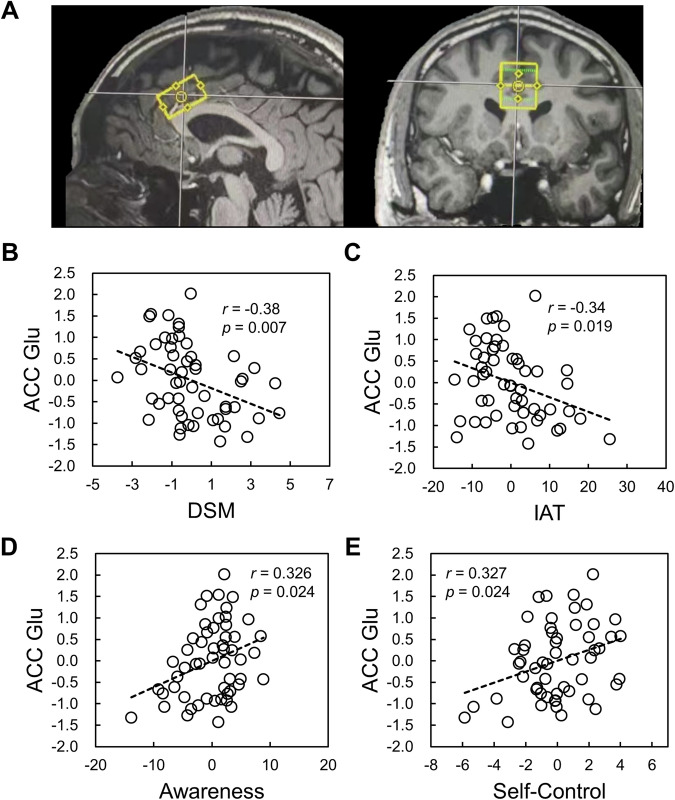
The relationship between glutamate concentration in dACC and self-reported characteristics. **A** The anatomic location of dACC where the MRS data were collected from. The partial correlation analyses showed that glutamate concentration in dACC was negatively correlated with DSM (**B**) and IAT (**C**), and positively correlated with mindful awareness (**D**) and self-control (**E**).

The relationship between glutamate concentration in PrL and drug seeking in rats implies the inability of regulating and inhibiting the addictive-like drug use behavior. However, the functional implications of prefrontal glutamate in addictive behavior are still elusive, which undermines the translational value to further understand the neuropsychological mechanisms of addiction. To this end, we assessed the glutamate concentration using MRS in human dACC (Fig. 4A), and further investigate it’s relationships with IGD severity, self-control, and mindful awareness level.

The partial correlation analyses revealed that mindful awareness and self-control were negatively correlated with severity of IGD, assessed with both IAT and DSM (Table 1). Noting that though the correlation between IAT and DSM scores are significantly positive, the effect size is moderate [47]. The two measures may capture different aspects of IGD and, therefore, were not merged.

**Table 1:** Partial correlations between behavioral measurements.

	IAT		Awareness		Self-Control	
	*r*	*p*	*r*	*p*	*r*	*p*
DSM	0.52	<0.001^**^	−0.36	0.010^*^	−0.34	0.017^*^
IAT	-	-	−0.63	<0.001^**^	−0.46	0.001^**^
Awareness	-	-	-	-	0.6	<0.001^**^
Self-Control	-	-	-	-	-	-

Covariates: age, gender, level of education, SMD.

*IAT* internet addiction test, *SMD* social media disorder.

**p < 0.01; *0.01 ≤ *p* < 0.05.

Consistent with our hypothesis, dACC glutamate concentration was found to negatively correlate with severity of IGD assessed using DSM (*r* = −0.38, *p* = 0.007, Fig. 4B) and IAT (*r* = −0.34, *p* = 0.019, Fig. 4C), controlling for multiple confounds (see methods). Further, the dACC glutamate concentration was positively correlated with mindful awareness (*r* = 0.326, *p* = 0.024, Fig. 4D) and self-control (*r* = 0.327, *p* = 0.024, Fig. 4E). Further analysis indicated that the dACC glutamate concentration was positively correlated with “resistant to temptation” subscale of self-control, which may reflect “regulation of craving/temptation”. The brain-behavioral correlation is not significant for “healthy habit” (Supplementary Fig. 3), suggesting the association between dACC glutamate concentration and behavior has specificity. Of note, we also measured the glutamate concentration in V1 as a control and found that it was not correlated with any of IAT, DSM, self-control, or mindful awareness (Supplementary Fig. 2). These relationships remain non-significant when using the same participants for the dACC region (Supplementary Fig. 4).

**Fig. 5:**
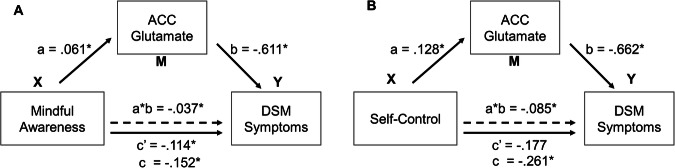
Mediation models considering the mediation effect of glutamate concentration in dACC. **A** Glutamate concentration in dACC partially mediated the relationship between mindful awareness and DSM. **B** Glutamate concentration of dACC completely mediated the relationship between self-control and DSM.

To investigate whether individual’s glutamate concentration in dACC mediates the relationship between the level of mindful awareness and addictive behavior, we constructed two mediation models, with DSM symptoms and IAT scores serving as dependent variables, respectively. This selection of dependent and independent variables aligns with established mediation analysis principles, which recommend selecting a more stable or causally prior variable as the independent variable to more accurately delineate the underlying mechanisms of observed effects [48]. The results revealed a partial mediation effect of dACC glutamate on the relationship between mindful awareness and DSM symptoms (Fig. 5A). Specifically, a significant total effect of mindful awareness on addictive severity measured by DSM was identified (c = −0.152, *p* = 0.007). The mediation effect of glutamate concentration was significant (a*b = −0.037, CI [−0.083, −0.004]), and the direct effect of mindful awareness on DSM was also significant (c’ = −0.114, *p* = 0.044). However, the mediation effect of glutamate concentration on the relationship between mindful awareness and IAT was not significant (a*b = −0.091, CI [−0.280, 0.061]).

Similarly, to investigate whether individual’s glutamate concentration in dACC also mediates the level of self-control and addictive behavior, we further constructed two mediation models, with DSM symptoms and IAT scores serving as dependent variables, respectively. The results revealed a total mediation effect of dACC glutamate on the relationship between self-control and DSM symptoms (Fig. 5B). Specifically, the total effect of self-control on DSM was significant (c = −0.261, *p* = 0.029), as well as the mediation effect (a*b = −0.085, CI [−0.203, −0.007]). The direct effect of self-control on DSM (c’ = −0.177, *p* = 0.140) was not significant. However, the mediation effect of glutamate concentration on the association between self-control and IAT was not significant (a*b = −0.276, CI [−0.739, 0.171]).

## Discussion

In the present study, we investigated the relationship between neurotransmitter concentration in PrL/dACC and addiction behavior. Using an animal model, we demonstrated that the glutamate concentration in PrL (the rodent homolog of human dACC) was significantly lower in METH group relative to SAL group after a 20-day SA, and the glutamate concentration change from pre-training baseline to post-punishment was negatively correlated with drug seeking behavior. Similarly, the glutamate concentration in dACC was negatively correlated with severity of IGD assessed using DSM and IAT in humans, whereas its correlations with self-control and mindful awareness were significantly positive. Further analyses of the clinical data revealed that glutamate concentration of dACC mediated the relationship between self-control/mindful awareness and DSM-based IGD severity. As discussed below, these results provide novel insights to the role of dACC in addiction.

### The chain of anticipation, seeking and taking in the recurrence of addictive behavior

The recurrence of addictive behavior follows a chain of anticipation, seeking, and taking, with cognitive control potentially influencing each of these stages [49, 50]. Anticipation or craving is a psychological process that can occur independent from the presence of the addictive substance or behavioral reinforcement [51]. Seeking behavior is motivated by anticipation [52], while taking behavior involves the pharmacological effects of the substance in SUD or the reinforcing aspects in cases of IGD.

In our animal model, we observed a correlation between total drug-infusion and cue-elicited seeking behavior at abstinence day 3, indicating that seeking contributes to drug abuse. However, the reduction of drug infusion under punishment relative to that immediately prior to the punishment did not correlate with cue-elicited seeking at abstinence day 3. These findings suggest that the impact of punishment on drug taking may involve additional factors beyond cue-induced seeking. Although the initial drug use is an essential condition for craving, the intensity of craving can vary greatly among individuals exposed to the same drug dose. Stronger craving may potentiate drug-seeking behavior, leading to increased drug taking. Therefore, we speculate that seeking behavior may contribute to drug use disorder independent from the combined effects of reward and punishment.

It is worth noting that in our animal model, trials involving punishment in the form of shocks were alternated with non-punishment trials. This schedule allows some rats to continue drug use despite punishment, manifesting compulsive behavior, while others nearly stop, which would result in a larger dynamic range facilitating correlational analysis of brain-behavioral association. However, in human addiction, punishment is typically not administered as closely in time to the rewarding experience as in the animal model. Although the animal model could not fully capture human addiction’s compulsive nature, this is probably one of the best available rodent models as animals may lack the cognitive ability to associate delayed punishment with drug-taking. In the case of IGD in humans, the behavior is often manifested through occupation and engagement in gaming without immediate concurrent punishment. As a result, mindful awareness of one’s behavior becomes crucial in regulating IGD behavior by detecting and inhibiting seeking impulses, thereby preventing abuse. As discussed in sections below, the monitoring function of the dACC/PrL plays a vital role in this process.

### Decrease of PrL glutamate concentration after self-administration may associate with impaired functionality of the region

The decrease of glutamate concentration in PrL after SA aligns with previous studies [53, 54] and provides additional insight into the changes in glutamate levels in the region during the course of addiction. Interpreting the functional implications of changes in glutamate levels measured by MRS is challenging, as these measurements can be affected by various factors including synaptic glutamate levels, the number of glutamatergic synapses, and extra-synaptic glutamate levels involved in neurometabolism [55–57]. However, based on the following considerations, we argued that the lower PrL glutamate concentration in the METH group after SA may relate to impaired functionality of this brain region. First, previous research has demonstrated a high correlation between neurotransmitter and metabolic pools [58, 59], with glial and extracellular glutamate pools being much smaller than the neuronal pool [60]. Second, the glutamate concentration in ACC was positively correlated with synaptic vesicle glycoprotein, a protein selectively expressed in presynaptic terminals, suggesting that glutamate levels measured by MRS likely reflect the concentration of glutamate in the presynaptic neurotransmitter pool [57, 61]. Third, our previous study [13] and work from others [62–65] have demonstrated a positive correlation between task-induced activation and regional glutamate concentration. Based upon above findings, the decrease of glutamate concentration could be a neurochemical mechanism underlying the hypoactivation of dACC seen in human addicts [3, 66].

Regarding the reasons for the lower glutamate concentration in the METH group after SA, a previous study has shown that chronic binge drinking could result in aberrant synaptic pruning and significant loss of excitatory synapses in the prefrontal cortex [67]. It is possible that synaptic loss occurs following drug use, resulting in a lower glutamate concentration. However, further investigations are necessary to validate this hypothesis.

### Inverse correlation between PrL glutamate concentration and drug seeking behavior may indicate weakened cortical-striatal regulation

In the current study, we found a significant negative correlation between the glutamate concentration in PrL after punishment and drug seeking behavior. This finding is consistent with a previous study showing that individuals who failed to resist the urge of smoking exhibited significant lower glutamate level in dACC compared with those successfully maintained subsequent abstinence [68]. Previous studies have identified that the glutamatergic projection from prefrontal cortex to nucleus accumbens, a critical region of the reward system, plays an important role in drug relapse [69]. The ventral striatum consists of two types of medium spiny neurons (MSNs), namely D1 and D2 MSNs, with the former relating to motivational promoting and the latter relating to motivational inhibition [70] and both receive glutamatergic input from the cortex. Combined with previous findings that the rsFC between PrL/dACC and ventral striatum was negatively correlated with compulsive drug taking behavior [10] and internet addiction symptoms [17], we can conjecture that lower glutamate concentration in dACC may further reduce the excitability of the projection from prefrontal cortex to D2 MSNs of ventral striatum and, therefore, weaken the control over craving.

Of note, glutamate concentration in PrL after punishment was only correlated with acute drug-seeking behavior but not with prolonged drug-seeking behavior. This result suggests that drug-seeking behavior after long term abstinence is different from that of acute abstinence during which the residual pharmacologic effect of METH may still contribute to the seeking motivation. We also found that the glutamate changes from pre-training baseline session to the punishment session was negatively correlated with drug seeking behavior in both acute and prolonged abstinence, suggesting the plasticity of PrL excitability after experiencing both reward and punishment may predict drug seeking behavior in a stable way less sensitive to abstinence status, and is a reliable biomarker for relapse prediction.

Furthermore, our findings indicate that the concentration of glutamate in the PrL is specifically associated with drug-seeking behavior rather than compulsive drug-taking behavior. This suggests that the PrL may be more involved in regulating anticipation and craving, rather than directly influencing the actual consumption of substances or engagement in compulsive behaviors. It is possible that the PrL’s contribution in regulating compulsive drug taking behavior is diminished due to the increased influence of its opponent affective system at the presence of substance.

### Negative/positive correlations between dACC glutamate concentration and addictive/ self-disciplined behaviors highlight the role of dACC excitability in self-initiated behavioral control

Self-control, which enables us to monitor and override reflexive and habitual reactions in order to achieve long-term goals, is essential for daily life and occupational success [29, 71]. Considering self-control in the addiction cycle also plays a significant role in understanding the development of addiction. In line with the animal experiment, the current human study showed a negative correlation between glutamate concentration in dACC and the severity of IGD. This further supports the idea that glutamate concentration in the dACC may serve as a potential neurochemical mechanism modulating the relationship between self-control and addictive behaviors, as confirmed by the mediation analyses.

A previous research has reported that individuals with low monitoring performance exhibit weaker functional connectivity between dACC and frontoparietal control network during rest [72]. In trials involving a high level of conflict, the dACC shows significant activation, and its activity can predict behavioral adjustment on the subsequent trials [73]. For internet addicts, significant impaired error-monitoring, inhibitory control, and abnormal high impulsivity have been identified, along with abnormal activity in the dACC [74–76]. These results collectively indicate that during the trajectory from recreational to impulsive/compulsive internet use, the ability to be aware of present-moment experience and monitor the conflict between current behavior or intention and long-term goals is critical for subsequent behavior adaptation [77]. Deficits of these cognitive control processes may contribute to the development of addictive behaviors. Our results highlight the role of glutamate concentration in dACC in such essential processes modulating the self-initiated behavioral control.

### The mediation role of dACC glutamate concentration in the relationship between mindful awareness and addictive behavior shields light to the development of a unified prevention and therapeutic strategy targeting the dACC functionality

While both IAT and DSM are valuable tools and have been widely used in previous studies for assessing the severity of internet gaming disorder [78–82], they capture slightly different aspects of the disorder. The IAT measures a broader spectrum of internet-related behaviors and their impact on daily life, which includes but is not limited to gaming. On the other hand, the DSM criteria are specifically focused on the diagnostic aspects of internet gaming disorder, adhering strictly to the clinical symptoms outlined in the manual.

In our study, the negative correlations of glutamate levels in dACC with both IAT and DSM scores suggests a general relationship between glutamate concentration and excessive use of internet. However, the mediation effect being significant only in the model with DSM as the dependent variable might indicate that the DSM’s more specific criteria align more closely with the neurochemical changes we observed. This insight underlines the importance of considering both general and specific measures when studying the neurobiological underpinnings of behavioral disorders.

Given the serious consequences of SUD and behavioral addition such as IGD, it is crucial to develop effective and non-invasive interventions for its prevention and treatment, benefiting both individuals and society. Mindfulness meditation, characterized by non-judgmental attention to present-moment experiences [83, 84], can be subdivided into focused attention and open monitoring approaches [85]. Mindfulness training is believed to leverage cognition through increased self-awareness [86]. Indeed, practitioners of mindfulness reported enhanced awareness of bodily states and improved perceptual clarity of subtle interoception after mindfulness practice [87]. Moreover, both longitudinal and cross-sectional studies have demonstrated improved conflict monitoring in individuals engaged in mindfulness training [87, 88]. These results indicate the potential for mindfulness as an intervention for preventing or treating addiction [89].

Supporting this notion, research by Tang et al. found that two weeks of meditation training resulted in a significant 60% reduction in smoking and increased activity of brain regions related with cognitive control, including the dACC [90, 91]. Beyond substance addictions, mindfulness training also reduces levels of gambling severity, gambling urges, and emotional distress in problem gamblers [92]. In the present study, we found that the glutamate concentration in the dACC mediated the relationship between mindful awareness and internet IGD symptoms. This suggests that mindfulness training targeting self-awareness may serve as a potential intervention for preventing IGD, with the changes in glutamate levels in the dACC potentially serving as biomarkers for assessing the intervention effects.

The study was not without its limitation. First, the animal experiment would benefit from integration of more comprehensive metrics, such as increased response requirements, progressive ratio, resistance to extinction, punishment, and robust incubation of craving, to better proxy human addiction severity. Second, although the population from which we recruited participants had a relatively low prevalence of tobacco and alcohol use, the lack of control for these factors may still influence the results. Third, only male rats were used in the current study, which limits the generalizability of the findings.

## Conclusion

Our study investigated glutamate concentration in the prefrontal cortex (PrL/dACC) and its relationship to addictive behaviors using both animal and human data. In the animal model, we observed lower glutamate concentration in the PrL after drug self-administration in the METH group relative to the SAL controls. This glutamate concentration was negatively correlated with drug seeking behavior, indicating a potential role of prefrontal glutamate in addiction. Similarly, in our human study, we observed a negative correlation between dACC glutamate concentration and the severity of IGD. Notably, glutamate concentration mediated the relationship between self-control/mindful awareness and IGD symptoms. These findings highlight the importance of the dACC in addiction development and suggest that interventions enhancing dACC activity, like mindfulness training, may have the potential to prevent addiction. Over all, this study provides valuable insights into the neurochemical mechanisms of addiction and offers translational potential in developing effective interventions for addictive disorders.

## Supplementary information

supplementary materials

## Data Availability

The data used to generate results of this study are available from the corresponding author upon reasonable request.
