# Supplementary material for: Glutamate concentration of medial prefrontal cortex is inversely associated with addictive behaviors: a translational study
Source: Transl Psychiatry. 2024 Oct 12;14:433. doi: 10.1038/s41398-024-03145-x (PMC11470925; doi:10.1038/s41398-024-03145-x)
Supplement: Supplementary file 1 — supplementary materials [file 41398_2024_3145_MOESM1_ESM.docx]

**Glutamate Concentration of Medial Prefrontal Cortex is Inversely Associated with Addictive Behaviors: A Translational Study**

**Running title: A Translational Study of Addiction**

Hui Zhou, Tiantian Hong, Xi Chen, Conghui Su, Binyu Teng, Wan Xi, Jean Lud Cadet, Yihong Yang, Fengji Geng, Yuzheng Hu

**^Supplementary Materials^**

Contents

[Supplementary methods 1](#_Toc177729261)

[Surgery of animals 1](#_Toc177729262)

[Self-administration training and foot shock punishment 1](#_Toc177729263)

[Abstinence and cue-reactive tests 2](#_Toc177729264)

[Questionnaire assessments in human study 2](#_Toc177729265)

[IAT 2](#_Toc177729266)

[DSM-5 3](#_Toc177729267)

[Five-Facet Mindful Questionnaire 3](#_Toc177729268)

[Self-control Scale 4](#_Toc177729269)

[SMD 4](#_Toc177729270)

[BSI 4](#_Toc177729271)

[MRI experiments 5](#_Toc177729272)

[Animal preparation 5](#_Toc177729273)

[Supplementary Table 1 6](#_Toc177729274)

[Supplementary Figure 1 7](#_Toc177729275)

[Supplementary Figure 2 8](#_Toc177729276)

[Supplementary Figure 3 9](#_Toc177729277)

[Supplementary Figure 4 10](#_Toc177729278)

[Supplementary Table 2 11](#_Toc177729279)

# Supplementary methods

## Surgery of animals

Under ketamine and xylazine (50 and 5 mg/kg, i.p., respectively) anesthesia, silastic catheters were surgically inserted into the jugular vein, following previously established procedures (1, 2). The catheters were connected to a modified 22-gauge cannula placed beneath the skin of the back between the shoulder blades. To maintain catheter patency, they were flushed every 24-48 hours with a solution containing gentamicin (Butler Schein; 5 mg/ml) and sterile saline. Additionally, buprenorphine (0.1 mg/kg, s.c.) was administered post-surgery to alleviate any pain experienced by the rats. A recovery period of 7 days was provided before commencing METH SA (self-administration) training.

## Self-administration training and foot shock punishment

The SA chambers were located inside sound-attenuating cabinets that were controlled by a Med Associates system (Med Associates, St Albans, VT, USA). Each chamber was equipped with two levers: an active lever and an inactive lever. Pressing the retractable active lever triggered an infusion pump, delivering either METH or saline. Pressing the inactive lever had no reinforced consequences.

The SA training procedure has also been previously described (1, 2). In brief, the rats were trained to self-administer dl-methamphetamine HCl, obtained from the pharmacy of National Institute on Drug Abuse (NIDA), at a dose of 0.1 mg/kg/infusion over a duration of 3.5 s. Alternatively, saline was administered during three, 3-h sessions/day, with each separated by a 30-minute interval, for a total of 20 days. This training occurred under a fixed-ratio (FR)-1 reinforcement schedule with a 20-s timeout. A 5-s compound tone-light cue was paired with each infusion. To minimize weight loss, the rats were trained in four cycles of 5 days on and 2 days off. After the 20-day SA training period, a 5-day punishment phase was initiated. During this phase, pseudorandom foot shock (FS) lasting 0.5 seconds were introduced alongside METH SA, occurring with half of the reinforced lever-presses. The intensity of the foot shocks varied over the 5 days in a predetermined fixed order (0.18, 0.24, 0.3, 0.3, and 0.3mA) (1, 2). It is important to note that a subset of eight METH rats and four SAL rats underwent a longer FS period than 5 days. Consequently, the data collected from these rats were not included in any analyses pertaining to the data collected after the FS period, resulting 18 METH rats and 11 SAL rats in these analyses.

## Abstinence and cue-reactive tests

After the completion of the 5-day punishment phase, the rats were subjected to a 30-day period of forced abstinence. Cue-presentation (extinction) tests were conducted on day 3 and day 30 of the abstinence period, each lasting for 30 minutes (Figure 2A). During these sessions, the rats were placed back into the SA chambers, where pressing the previously active lever no longer resulted in the delivery of METH or saline. Instead, pressing the lever solely triggered the presentation of the tone-light cue that was previously associated with METH/saline infusions.

## Questionnaire assessments in human study

IAT **(Internet Addiction Test)**

The severity of internet addiction among participants was assessed using the Chinese version of internet addiction test (IAT) (3, 4), which consists of 20 items assessing the extent to which internet use affects individuals’ daily routine, social life, productivity, sleeping pattern, and feelings. Participants were asked to rate each item on a five-point Likert scale, where a score of one corresponds to “rarely” and a score of five corresponds to “always”. The higher the score, the more severe the symptom of internet addiction. The IAT questionnaire was confirmed to have good reliability and validity among Chinese adolescents (4).

DSM-5 **based Internet gaming disorder scale (abbreviated as** DSM)

The DSM scale was used to assess the severity of IGD (5). The scale consists of 9 criteria recommended by the DSM-5 (6), and was developed with a consensus of a group of prominent international experts (5). Participants were required to respond with a “yes” or “no” to each item according to their experiences in the last 12 months. Online gambling was excluded from the scale. To characterize IGD with multiple severity levels, we used the total number of 'yes' responses from participants as a proxy for IGD severity. The higher the score, the higher the IGD risk. The IGD scale was considered to have good reliability and validity (7).

### Five-Facet Mindful Questionnaire

The Chinese version of five-facet mindful questionnaire was conducted to measure the trait mindfulness of participants (8, 9). The scale consists of 39 items, which were divided into five subscales as follows: 1) *Observing*, the ability of noticing internal and external stimuli, including emotion, sensation, cognition, and visual perception. 2) *Describing*, the ability of noting and mentally identifying internal experiences with words. 3) *Acting with awareness*, the ability of keeping focus on the current activities. 4) *Non-judging of inner experience*, the ability of not evaluating one’s sensations, emotions, and cognitions. 5) *Non-reactivity to inner experience*, the ability of not being absorbed in inner thoughts and feelings. Participants were required to rate each item on a five-point Likert scale, ranging from one (“not at all”) to five (“exact match”). The score for each subscale was calculated as the sum of scores for all items belonging to this subscale. The scale was considered to have good reliability and validity (8), and the third subscale, *Acting with awareness*, was used as a measure of mindful awareness in the present study.

### Self-control Scale

The Chinese version of self-control scale was used to measure individuals’ level of self-control (10, 11). The scale consists of 19 items and participants were required to rate each item on a five-point Likert scale (“1” for “not at all” and “5” for “very much”). The 19 items were divided into 5 dimensions and the score for each dimension was calculated as the mean score of all items belonging to this dimension. The total score of self-control was determined as the sum of all dimension scores. The higher the score, the better the self-control ability. The self-control scale was considered to have good reliability and validity (10).

SMD **(****Social media disorder Scale)**

The Chinese version of social media disorder scale was conducted to measure the problematic usage of social media, which is based on DSM criteria and consists of 9 items (6, 12). Participants were required to respond with a “yes” or “no” to each item according to their experiences in the past 12 months. The social media here was defined as the most popular social networking site applications in China, such as WeChat, Sina Weibo, Tencent Weibo, and Douyin. The higher the score, the higher the risk of social media addiction. The scale was considered to have good reliability and validity in Chinese adolescent (12). As internet game and social media are the primary two types of recreational use of Internet, the SMD measure, which has not been formally considered as a mental disorder/condition, was used as a covariate when investigate IGD.

BSI **(Brief symptom inventory)**

The Chinese version of the anxiety and depression dimensions in the BSI (13) were used to assess the severity of anxiety and depression. Each dimension consists of six items. Participants were required to rate each item on a five-point Likert scale, ranging from one (“not at all”) to five (“extremely”). A higher total score indicates a more severe symptom.

## MRI experiments

Animal preparation

For magnetic resonance imaging (MRI) scans, the animals were anesthetized using a combination of isoflurane (Piramal Critical Care Inc., Bethlehem, PA, USA) and dexmedetomidine hydrochloride (Zoetis Services LLC, Parsippany, NJ, USA), following a protocol that has been described previously (14). In brief, anesthesia was induced with 2% isoflurane and a subcutaneous bolus injection of dexmedetomidine at a dose of 0.02 mg/kg. The rats were securely positioned in an animal holder equipped with a bite bar, and their core body temperature was maintained at 37±1°C using a water-circulating heating pump. Continuous monitoring of heart rate and blood oxygenation levels was carried out using a noninvasive pulse oximeter attached to the hind foot of the animal. Additionally, respiration rate was monitored with a MouseOx sensor (Starr Life Sciences, Oakmont, PA, USA) positioned beneath the animal's chest. During the acquisition of functional MRI (fMRI) data, the respiration rate, oxygenation levels, and heart rate exhibited variations within the ranges of 65 to 80 cycles per minute, 90% to 100%, and 250 to 320 beats per minute, respectively.

# Supplementary Table 1

Supplementary Table 1. Demographic information of human participants (n = 54).

| Age (years) | Mean (SD) | 22.33 (2.42) |
| --- | --- | --- |
|  | Range | [18, 28] |
| Gender | Male | N = 35 |
|  | Female | N = 19 |
| DSM score for IGD | >=5 | N = 10 |
|  | Mean (SD) | 2.61 (2.14) |
| Gaming Time | 6-8h/day | N = 1 |
|  | 4-6h/day | N = 4 |
|  | 2-4h/day | N = 11 |
|  | 0-2h/day | N = 38 |
| Internet Addiction Test | Mean (SD) | 49.80 (12.67) |
| Self-control | Mean (SD) | 15.46 (2.56) |
| Social media disorder | Mean (SD) | 3.43 (2.11) |
| Mindful awareness | Mean (SD) | 27.02 (5.25) |
| Anxiety | Mean (SD) | 13.17 (4.76) |
| Depression | Mean (SD) | 10.78 (4.86) |

# Supplementary Figure 1


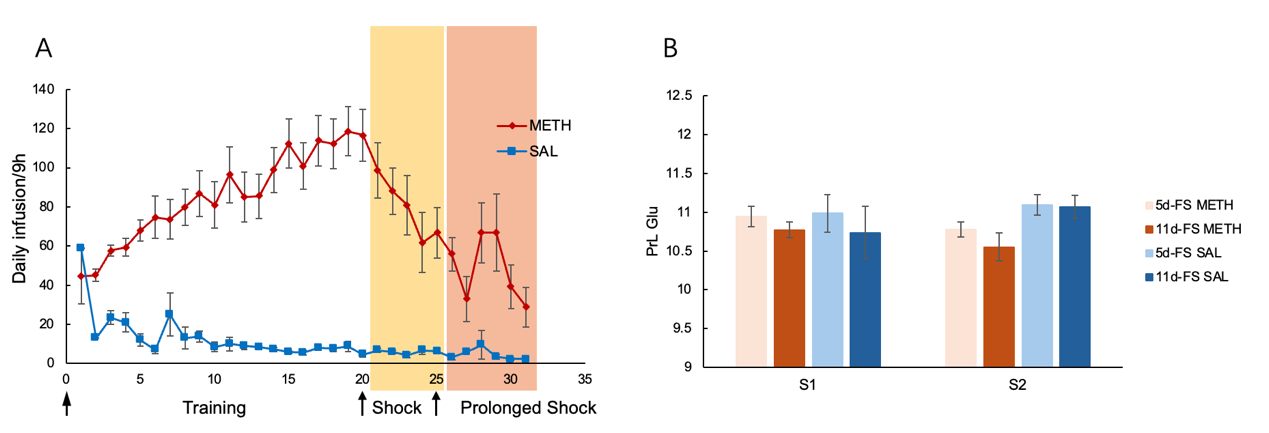


Supplementary Figure 1. The behavioral results and Glutamate levels of rats with longer FS period. (A) The METH rats underwent 11-day FS period increased their drug intake during the training phase and decreased when foot shock was introduced, whereas the SAL group remained at a low level of saline infusion. (B) The Glutamate levels of rats with longer FS period and with standard 5-day FS period. 5d-FS METH, METH rats underwent a 5-day foot-shock period; 11d-FS METH, METH rats underwent a 11-day foot-shock period; 5d-FS SAL, SAL rats underwent a 5-day foot-shock period; 11d-FS SAL, SAL rats underwent a 11-day foot-shock period. Error bars represents standard error of the mean.

# Supplementary Figure 2


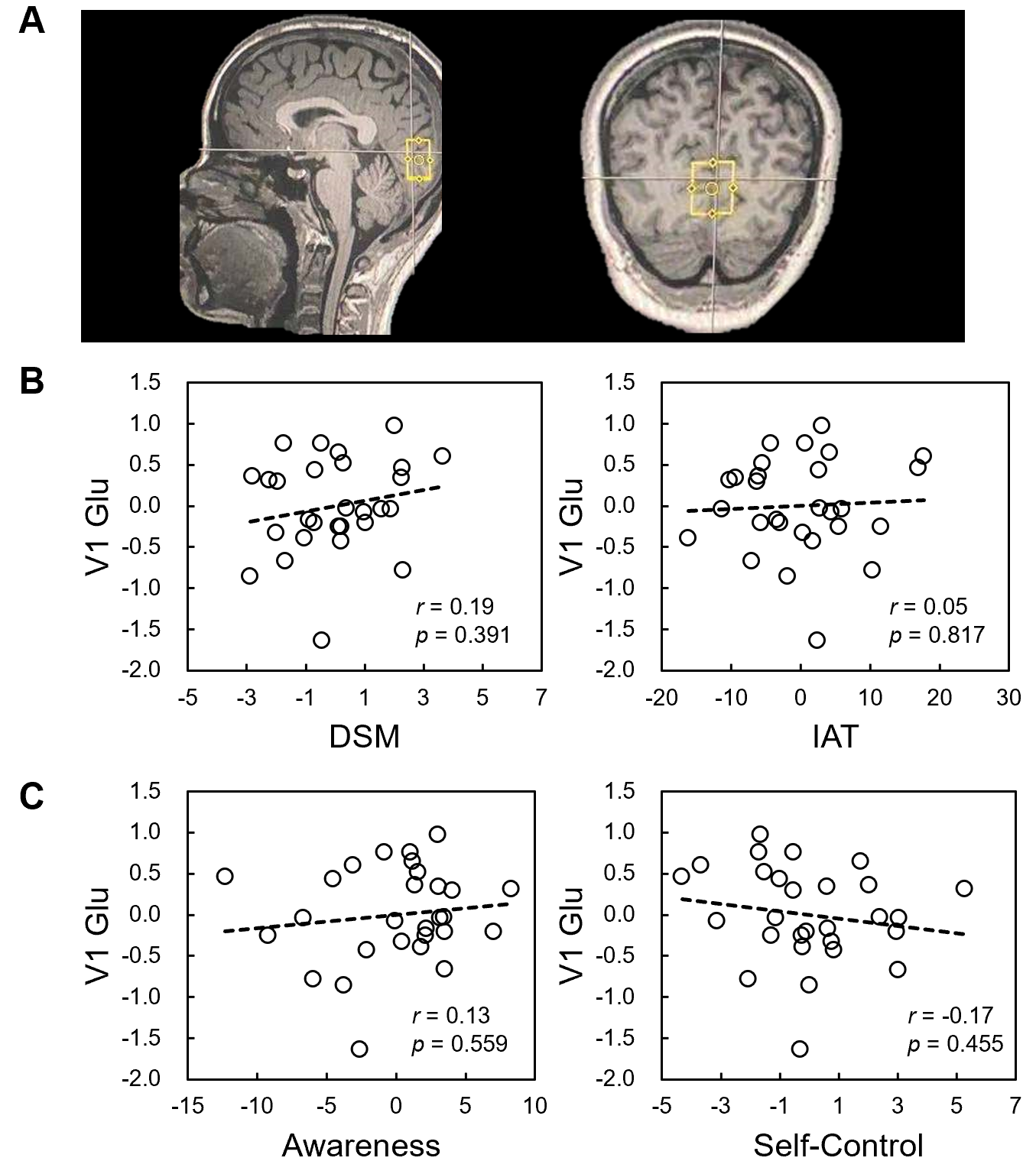


Supplementary Figure 2. Partial correlation suggests that the no significant correlation was found between glutamate concentration of V1 and DSM, IAT, mindful awareness, and self-control.

# Supplementary Figure 3


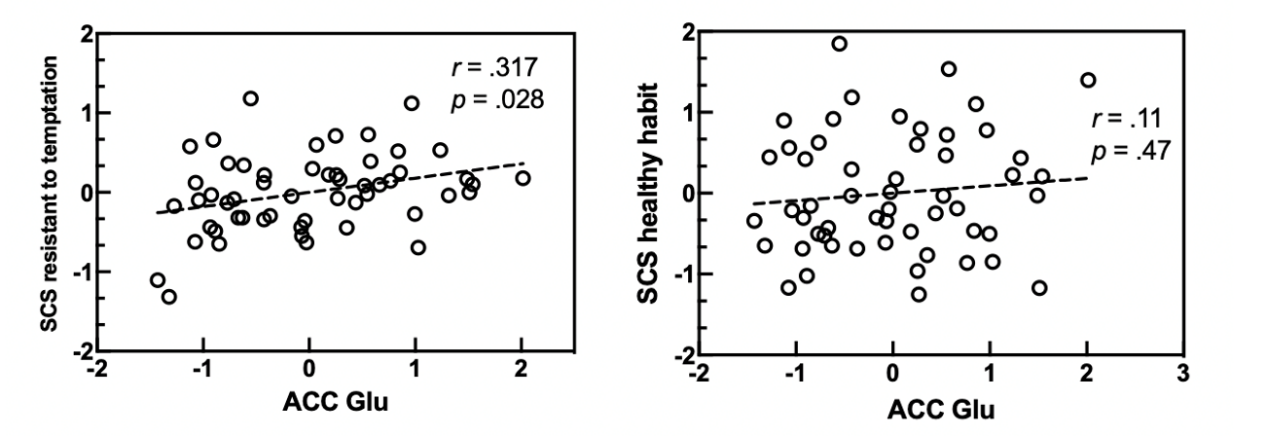


Supplementary Figure 3. Partial correlation between glutamate concentration of dACC and “resistance temptation”, “healthy habit” subscales of self-control.

# Supplementary Figure 4


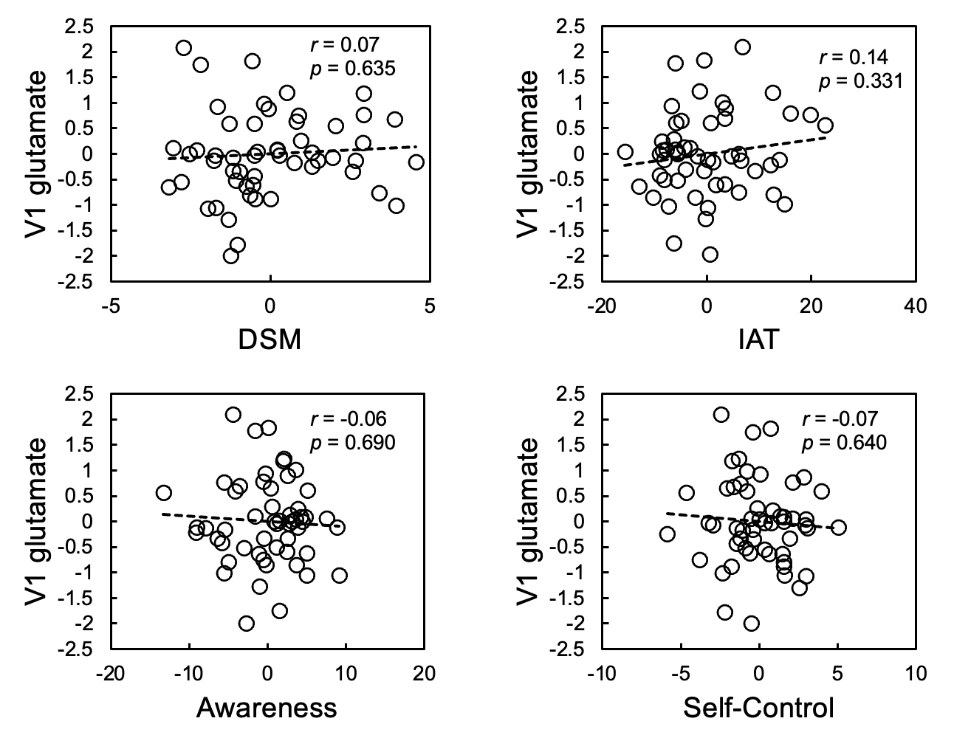


Supplementary Figure 4. Partial correlation plots for V1 (n = 54), with age, gender, education, CRLB, tissue components of CSF, and SMD (social media disorder scale) scores served as covariates.

# Supplementary Table 2

Supplementary Table 2. Skewness and kurtosis coefficients of glutamate levels

|  | Skewness  (standardized) | Kurtosis  (standardized) | Skewness | Kurtosis |
| --- | --- | --- | --- | --- |
| **Animal** |  |  |  |  |
| PrL Glu - session 1 - METH | 1.237 | 1.625 | 0.564 | 1.441 |
| PrL Glu - session 2 - METH | 0.544 | -0.893 | 0.248 | -0.792 |
| PrL Glu - session 3 - METH | -1.522 | 0.065 | -0.816 | 0.067 |
| PrL Glu - session 1 - SAL | -0.341 | -0.087 | -0.198 | -0.098 |
| PrL Glu - session 2 - SAL | -0.219 | -0.890 | -0.127 | -0.998 |
| PrL Glu - session 3 - SAL | -1.958 | 1.980 | -1.294 | 2.533 |
| **Human** |  |  |  |  |
| ACC glutamate | 1.212 | -1.426 | 0.394 | -0.911 |
| V1 glutamate | 1.563 | 0.056 | 0.508 | 0.036 |

Note: Datasets with standardized skewness and kurtosis coefficients within ±1.96 are considered to have a normal distribution (15, 16). Only PrL Glu at session 3 in the SAL group slightly violates the normality assumption.

Reference

1. J. L. Cadet, C. Brannock, I. N. Krasnova, S. Jayanthi, B. Ladenheim, M. T. McCoy *et al.*, Genome-wide DNA hydroxymethylation identifies potassium channels in the nucleus accumbens as discriminators of methamphetamine addiction and abstinence. *Mol Psychiatry* **22**, 1196-1204 (2017).

2. I. N. Krasnova, N. J. Marchant, B. Ladenheim, M. T. McCoy, L. V. Panlilio, J. M. Bossert *et al.*, Incubation of methamphetamine and palatable food craving after punishment-induced abstinence. *Neuropsychopharmacology* **39**, 2008-2016 (2014).

3. Laura Widyanto, Mary McMurran, The psychometric properties of the internet addiction test. *Cyberpsychology & behavior* **7**, 443-450 (2004).

4. Ching-Man Lai, Kwok-Kei Mak, Hiroko Watanabe, Rebecca P Ang, Joyce S Pang, Roger CM Ho, Psychometric properties of the internet addiction test in Chinese adolescents. *Journal of pediatric psychology* **38**, 794-807 (2013).

5. Nancy M. Petry, Florian Rehbein, Douglas A. Gentile, Jeroen S. Lemmens, Hans-Juergen Rumpf, Thomas Moessle *et al.*, An international consensus for assessing internet gaming disorder using the new DSM-5 approach. *Addiction* **109**, 1399-1406 (2014).

6. APA, *Diagnostic and statistical manual of mental disorders: DSM-5™, 5th ed*, Diagnostic and statistical manual of mental disorders: DSM-5™, 5th ed. (American Psychiatric Publishing, Inc., Arlington, VA, US, 2013), 10.1176/appi.books.9780890425596, pp. xliv, 947-xliv, 947.

7. Wei Lei, Kezhi Liu, Zhen Zeng, Xuemei Liang, Chaohua Huang, Ke Gong *et al.*, The psychometric properties of the Chinese version internet gaming disorder scale (vol 160, 106392, 2020). *Addict. Behav.* **113** (2021).

8. Yu-Qin Deng, Xing-Hua Liu, Marcus A. Rodriguez, Chun-Yan Xia, - The Five Facet Mindfulness Questionnaire: Psychometric Properties of the Chinese Version. **- 2**, - 128 (2011).

9. R. A. Baer, G. T. Smith, J. Hopkins, J. Krietemeyer, L. Toney, Using self-report assessment methods to explore facets of mindfulness. *Assessment* **13**, 27-45 (2006).

10. T. A. N. Shuhua, G. U. O. Yongyu, Revision of Self-Control Scale for Chinese College Students. *Chinese Journal of Clinical Psychology* **16**, 468-470 (2008).

11. J. P. Tangney, R. F. Baumeister, A. L. Boone, High self-control predicts good adjustment, less pathology, better grades, and interpersonal success. *Journal of Personality* **72**, 271-324 (2004).

12. Sai-fu Fung, Cross-cultural validation of the Social Media Disorder scale. *Psychology Research and Behavior Management* **12**, 683-690 (2019).

13. Leonard R Derogatis, Nick Melisaratos, The brief symptom inventory: an introductory report. *Psychological medicine* **13**, 595-605 (1983).

14. H. Lu, Q. Zou, H. Gu, M. E. Raichle, E. A. Stein, Y. Yang, Rat brains also have a default mode network. *Proceedings of the National Academy of Sciences of the United States of America* **109**, 3979-3984 (2012).

15. Hae-Young Kim, Statistical notes for clinical researchers: assessing normal distribution (2) using skewness and kurtosis. *Restorative dentistry & endodontics* **38**, 52 (2013).

16. Süleyman DemİR, Comparison of Normality Tests in Terms of Sample Sizes under Different Skewness and Kurtosis Coefficients. *International Journal of Assessment Tools in Education* **9**, 397-409 (2022).
